# Comparative Proteomic Analysis Provides Insights into the Regulatory Mechanisms of Wheat Primary Root Growth

**DOI:** 10.1038/s41598-019-47926-7

**Published:** 2019-08-13

**Authors:** Le Li, Yanhua Xu, Yongzhe Ren, Zhanyong Guo, Jingjing Li, Yiping Tong, Tongbao Lin, Dangqun Cui

**Affiliations:** 1 College of Agronomy, Henan Agricultural University, Zhengzhou, China; 2 State Key Laboratory of Wheat and Maize Crop Science, Henan Agricultural University, Zhengzhou, China; 3 Collaborative Innovation Center of Henan Grain Crops, Henan Agricultural University, Zhengzhou, China; 4 Shangqiu Normal University, Shangqiu, China; 5 State Key Laboratory for Plant Cell and Chromosome Engineering, Institute of Genetics and Developmental Sciences, Chinese Academy of Sciences, Beijing, China

**Keywords:** Proteomics, Plant molecular biology

## Abstract

Plant roots are vital for acquiring nutrients and water from soil. However, the mechanisms regulating root growth in hexaploid wheat remain to be elucidated. Here, an integrated comparative proteome study on the roots of two varieties and their descendants with contrasting root phenotypes was performed. A total of 80 differentially expressed proteins (DEPs) associated with the regulation of primary root growth were identified, including two plant steroid biosynthesis related proteins and nine class III peroxidases. Real-time PCR analysis showed that brassinosteroid (BR) biosynthesis pathway was significantly elevated in long-root plants compared with those short-root plants. Moreover, O_2_.^−^ and H_2_O_2_ were distributed abundantly in both the root meristematic and elongation zones of long root plants, but only in the meristematic zone of short-root plants. The differential distribution of reactive oxygen species (ROS) in the root tips of different genotypes may be caused by the differential expression of peroxidases. Taken together, our results suggest that the regulation of wheat primary root growth is closely related to BR biosynthesis pathway and BR-mediated ROS distribution.

## Introduction

Plant breeding is an important way to increase crop yield. Currently, breeding selection is mainly based on aerial traits, and genetic improvement for root traits is still relatively rare^1–4^. In many regions of the world, current crop production still depends on excessive application of fertilizer and water, which is detrimental to sustainable agricultural development^5–7^. Due to environmental pressures and resource shortages, improvements in resource use efficiency should be prioritized in future crop breeding programmes^8^. Roots are the main organs for plants to uptake nutrients and water. Genetic improvement of root traits is one of the important strategies to increase nutrient and water use efficiency of crops^2,7,9–11^. Therefore, it is important to study the molecular mechanisms that control the growth and development of plant roots in food crops to enable genetic improvement of root traits.

Previous studies in the model plant *Arabidopsis* demonstrated that plant root growth and development are regulated by phytohormones^12^. PLETHORA (PLT) transcription factors are indispensable to maintain the niche of root stem cell and function as principal regulators of the root apical meristem (RAM). The expression of *PLT* genes is induced by auxin accumulation^13,14^. Cytokinin controls root growth and development by balancing the division and differentiation of stem cells^15^. The balance between auxin and cytokinin in root tips plays a crucial role in determining the size of RAM^16^. The expression of *PLT* genes is also regulated by local brassinosteroids (BRs) signalling in the stele^17^. Mutants with weakened BR biosynthesis or signal transduction show a short-root phenotype^18^. Moreover, supplementing low concentration of BRs can promote root growth^19^.

Wheat (*Triticum aestivum* L.) is an important food crop. Several studies have reported a number of important quantitative trait loci (QTLs) for root traits in wheat^20–25^. He *et al*. conducted a proteomic study using 2D-DIGE (Two-Dimensional Difference Gel Electrophoresis) and identified 16 DEPs involved in the regulation of wheat root development^26^. Li *et al*. identified a plant-specific transcription factor, MORE ROOT (TaMOR). Overexpression wheat *TaMOR* gene in rice (*Oryza sativa*) results in larger root system and higher grain yield^27^. Another study showed that overexpression of a wheat NAC transcription factor gene can promote root growth and increase plant drought tolerance^28^. However, despite these advances, the molecular mechanisms controlling wheat root growth and development remain unclear.

Previously we developed a “XY54 × J411” recombinant inbred line (RIL) population. XY54 was the female parent of the RIL population and had significantly longer PRL (Primary root length) and MRL (Maximum root length) than the male parent J411^24,29^. In this study, fifteen long-root lines (Long root group, LR group) and another fifteen short-root lines (Short root group, SR group) were selected based on previous studies from the RIL population^24^. To minimize differences in genetic background, the SRM (Short Root Mixture, a root mixture of the SR group) and LRM (Long Root Mixture, a root mixture of the LR group), and the roots of XY54 and J411 were used as materials for comparative proteomic analysis to identify candidate regulatory proteins of wheat root growth. Differentially expressed proteins and significantly enriched pathways involved in the regulation of wheat primary root growth were identified. Further molecular and physiological analyses suggested that the regulation of wheat primary root growth is closely related to BR biosynthesis pathway and BR-mediated ROS distribution.

## Results

### Phenotype analysis of the LR and SR groups

**Figure 1:**
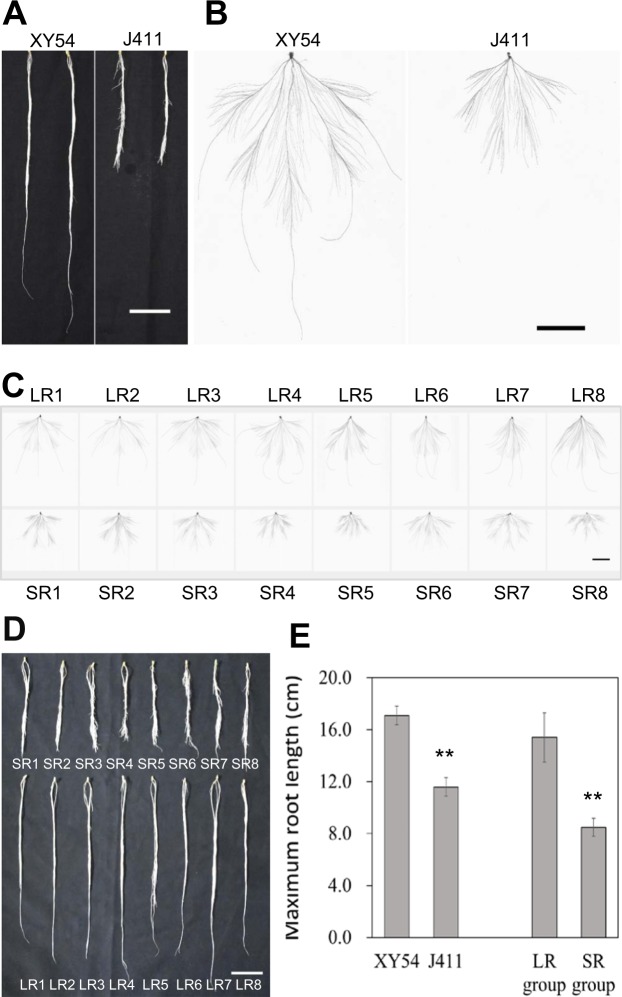
Root phenotype of wheat varieties XY54, J411, the LR group (Long root group) and the SR group (Short root group). (**A**, **B**) Roots (**A**) and root morphologies (**B**) of XY54 and J411; (**C**, **D**) root morphologies (**C**) and roots (**D**) of eight randomly selected lines from the LR group and SR group, respectively; (**E**) average values of MRL (maximum root length) of XY54, J411, LR group and SR group. The asterisks above the columns indicate significant differences at p < 0.05 (n = 15). MRL, maximum root length. Bar = 5 cm.

In this study, XY54 had significantly longer PRL and MRL than J411, which is consistent with our previous studies^24,29^ (Fig. 1A,B). Similarly, the PRL and MRL of the lines in the LR group were significantly greater than those of the lines in the SR group (Fig. 1C,D). The MRL of lines in the LR group varied from 13.6 cm to 19.4 cm, while the MRL of lines in the SR group varied from 7.2 cm to 9.4 cm. On average, the MRL of the LR group was 81.2% longer than that of the SR group (Fig. 1E).

### Identification and quantitative results of differential proteins

To identify proteins regulating wheat primary root growth, root protein profiles of XY54, J411, LRM and SRM were obtained. In total, we identified 6294 reliable quantified proteins for the following DEPs analysis (False discovery rate, FDR ≤1%; detected in at least two biological replicates; Supplementary Table S1). We identified 723 proteins as DEPs in one to one comparison between XY54 and J411 based on their respective abundance in roots (Supplementary Table S2). Among the DEPs, 373 proteins were up-regulated and 350 proteins were down-regulated in the roots of XY54. We also identified 452 proteins as DEPs in the LRM-SRM comparison. Among them, 235 DEPs exhibited up-regulation and 217 DEPs showed down-regulation in the roots of LRM (Supplementary Table S3).

**Figure 2:**
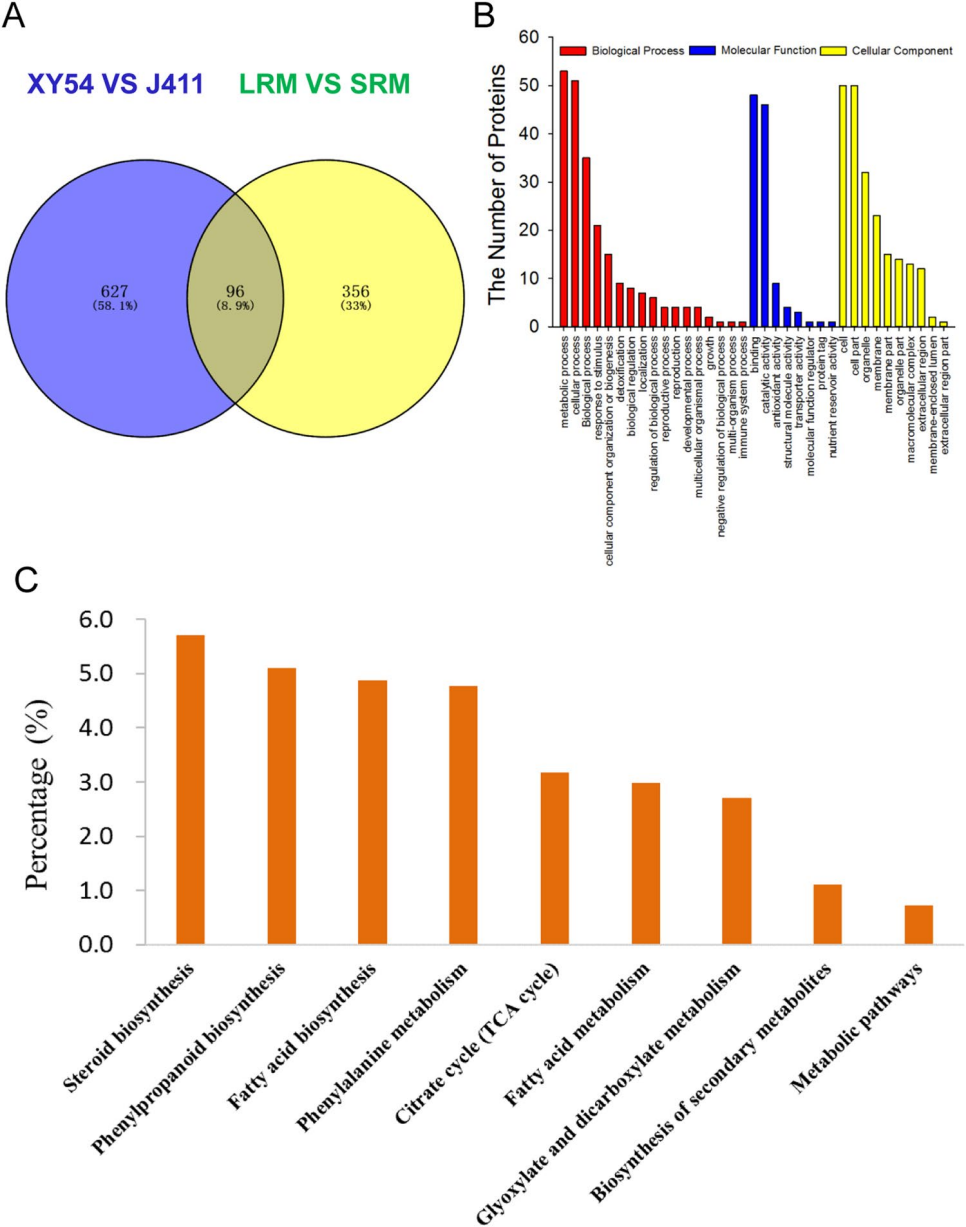
Results of Venn diagram analysis, and GO and pathway enrichment analysis of the DEPs. (**A**) Result of Venn diagram analysis; (**B**) GO enrichment analysis; (**C**) significantly enriched pathways involving the up-regulated DEPs. The values on the vertical ordinate represent the percentage of DEPs to the background proteins in the pathway. DEPs, differentially expressed proteins.

**Figure 3:**
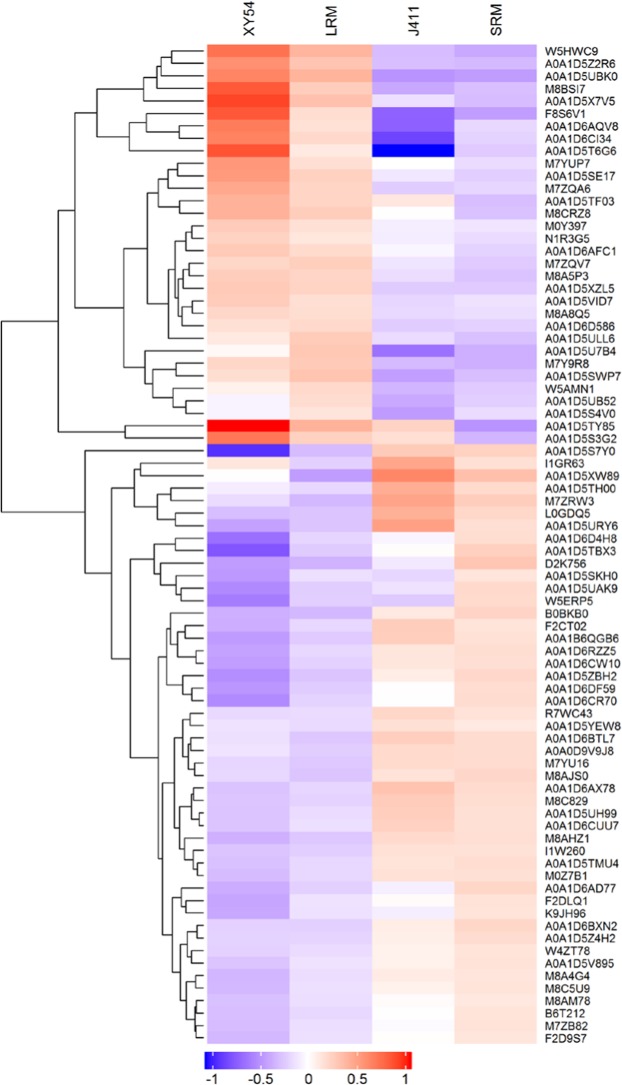
Hierarchical clustering of DEPs involved in the regulation of primary root growth. The four columns represent the protein expression levels in XY54, the long root mixture (LRM), J411 and the short root mixture (SRM). Red and blue indicate the higher and lower expression levels, respectively. DEPs, differentially expressed proteins.

Among the DEPs identified above, 96 existed in both the XY54-J411 comparison and LRM-SRM comparison (Fig. 2A). Of these 96 DEPs, 16 DEPs exhibited opposite expression patterns in the two subsets (up-regulated in one subset and down-regulated in the other), while the remaining 80 DEPs exhibited similar expression patterns (Supplementary Table S4). As described above, XY54 and lines of the LR group exhibited longer primary roots than J411 and lines of the SR group. Therefore, there is reason to believe that these 80 DEPs may be associated with PRL and MRL, because most of the differences in genetic background between the parents had been eliminated. The 80 DEPs that existed in both subsets are listed in Supplementary Table S4. Among them, 32 proteins were up-regulated and 48 proteins were down-regulated in the roots of XY54 and LRM, compared with J411 and SRM (Supplementary Table S4). Hierarchical cluster analysis of the 80 proteins showed that a distinct difference exists in abundance of these proteins between the long root genotype samples (XY54 and LRM) and the short root genotype samples (J411 and SRM). As expected, the 80 DEPs could be divided into two major distinct sub-clusters containing proteins with different levels of abundance (Fig. 3). According to the classification of cell component, molecular function and biological process, 80 DEPs were classified by GO (gene ontology) terminology. All the quantified protein annotations were taken as a background dataset, significantly enriched GO terms are shown in Fig. 2B (p < 0.05). Results showed that proteins involved in biological processes, such as the categories of metabolic process, cellular process, biological process and response to stimuli, were most common; while proteins associated with molecular functions were predicted to be involved in catalytic activity, antioxidant activity, binding, structural molecules, transport activity, molecular function regulation, nutrient reservoir activity and protein tags. Among all cellular components, ‘cell’ and ‘cell part’ were predicted to have the largest number of DEPs (Fig. 2B).

To gain insight into the DEPs involved in the regulation of PRL, we used the web-based software KOBAS 3.0 and identified nine significantly enriched pathways in the up-regulated DEPs in long rooted plants based on hypergeometric distributions (p < 0.05 after the FDR correction; Fig. 2C; Supplementary Table S5). Interestingly, plant steroid biosynthesis pathway was significantly enriched. There were two DEPs (one obtusifoliol 14-alpha demethylase and one delta (24)-sterol reductase) involved in plant steroid biosynthesis pathway (Fig. 2C). Obtusifoliol 14-alpha demethylase, encoded by the *CYP51* gene, catalyses the demethylation of obtusifoliol^30,31^. Delta (24)-sterol reductase, encoded by the *Dwarf1* (*DWF1*) gene, is involved in the formation of campesterol (CR), an early precursor of BRs^32^. Both CYP51 and DWF1 are essential for the biosynthesis of plant steroids. The expression of these two proteins was up-regulated in the roots of the LR group and long-root parent, XY54, compared with the SR group and short-root parent J411 (Fig. 3; Supplementary Table S4). Most DEPs in the phenylpropanoid biosynthesis pathway are peroxidases. In addition, the pathways for fatty acid biosynthesis and metabolism, phenylalanine metabolism and some carbon metabolism pathways (i.e. citrate cycle, glyoxylate and dicarboxylate metabolism) were also elevated in XY54 and LRM (Fig. 2C; Supplementary Table S5).

### Real-time PCR verification

**Figure 4:**
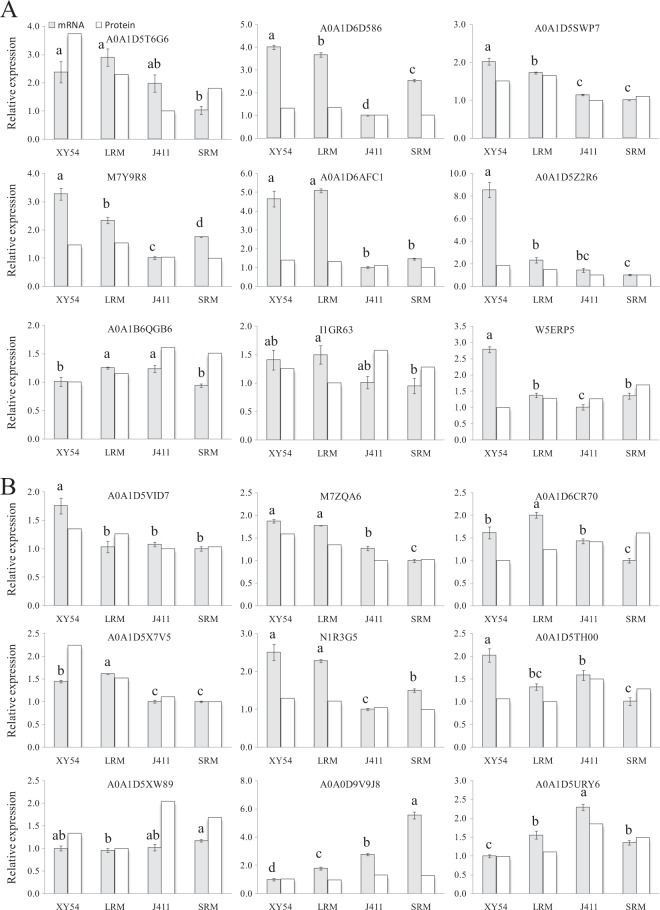
Relative mRNA expression analysis of DEPs using real-time PCR. (**A**) Relative mRNA expression of nine differentially expressed peroxidases; (**B**) relative mRNA expression of nine randomly selected DEPs. For the expression level of a protein, the lowest of the four samples was defined as a reference and its expression value was set as 1.0. Columns marked with different lowercase letters indicate that the difference between them (in gene expression levels) is significant according to Duncan’s multiple range test (n = 3, p < 0.05).

To evaluate the correlation of protein expression with their corresponding mRNA level, all nine differentially expressed peroxidases and another nine randomly selected DEPs were chosen for RNA level examination using real-time PCR (Fig. 4). Of the nine peroxidases genes, six genes closely matched their mRNA levels with their translation products and the other three genes (A0A1B6QGB6, I1GR63 and W5ERP5) had poor correlations between mRNA and protein expression levels (Fig. 4A). Of the nine randomly selected genes, five genes had closely matched mRNA levels with their translation products while the expression levels of the other four genes (A0A1D5VID7, A0A1D6CR70, A0A1D5TH00 and A0A1D5XW89) exhibited poor correlations with their changes in protein abundance (Fig. 4B).

### Real-time PCR analysis of BR biosynthesis related genes

**Figure 5:**
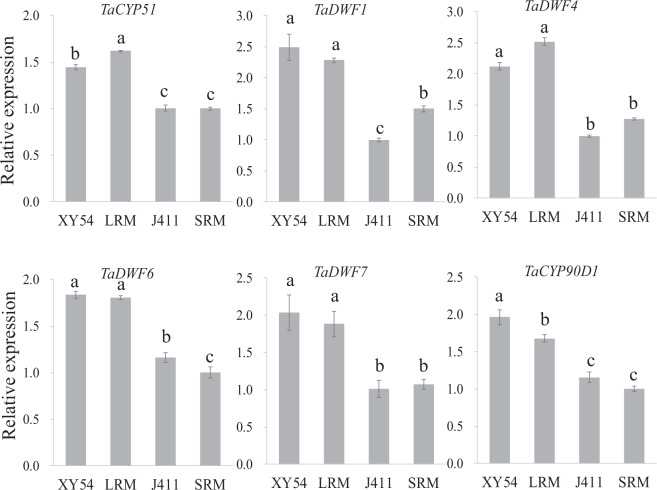
Relative mRNA expression analysis of genes involved in brassinosteroid biosynthesis using real-time PCR. The sample with the lowest expression level was set as 1.0. Columns marked with different lowercase letters indicate that the difference between them (in gene expression levels) is significant according to Duncan’s multiple range test (n = 3, p < 0.05).

CYP51 and DWF1 are two components involved in the biosynthesis of plant steroids. As both were significantly up-regulated in roots of the LR group and long-root parent XY54, compared with the SR group and short-root parent J411, we further checked the relative expression of *TaCYP51* and *TaDWF1*, and four other genes (*TaDWF4*, *TaDWF6*, *TaDWF7* and *TaCYP90D1*) involved in the BR biosynthesis pathway at an RNA level. Results showed that all of them were significantly up-regulated in XY54 and the LR group compared with J411 and the SR group (Fig. 5).

### *In Situ O*_*2*_.^-^ and H_2_O_2_ detection

**Figure 6:**
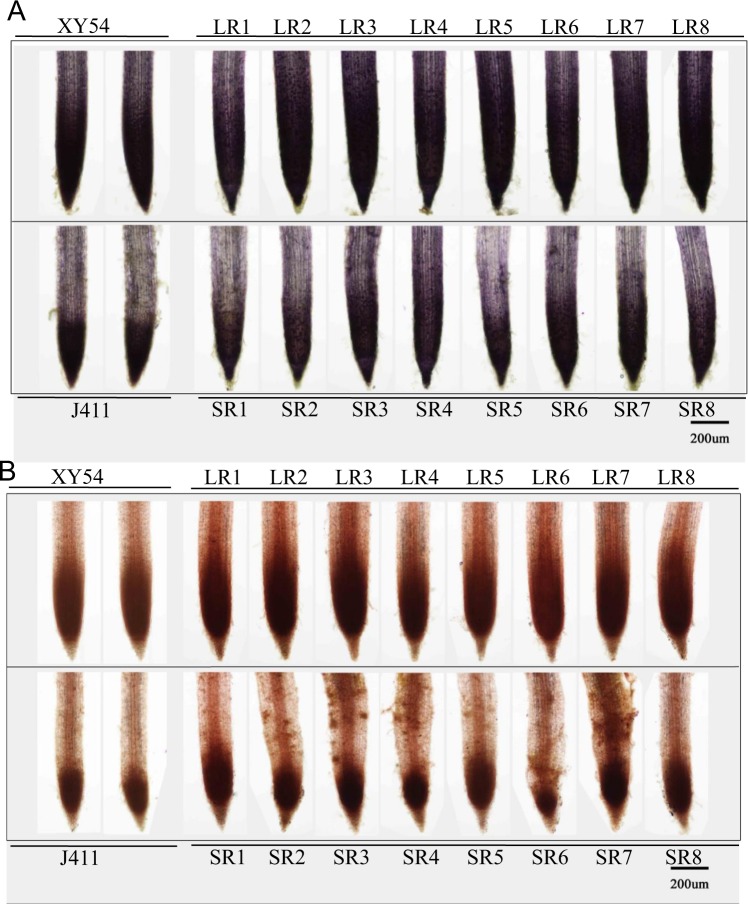
Results of *in situ* detection of root O_2_^.-^ and H_2_O_2_ in XY54, J411 and eight randomly selected lines from the long root (LR) group and short root (SR) group, respectively. NBT (Nitroblue tetrazolium) and DAB (3,3′-diaminobenzidine) were used to stain O_2_^.-^ and H_2_O_2_, respectively. (**A**) Results of NBT staining; (**B**) results of DAB staining.

As mentioned above, a significant number (nine) of peroxidases were present in the DEPs and most of them also exhibited differential expression at an RNA level (Fig. 4A). As peroxidases regulate the balance of ROS between the meristematic and elongation zones^33^, we further analysed the distribution of ROS (O_2_.^-^ and H_2_O_2_) in the root tips by staining roots with nitroblue tetrazolium (NBT) and 3,3′-diaminobenzidine (DAB), respectively; these reagents are widely used as indicators of O_2_.^-^ and H_2_O_2_ levels^34,35^. As shown in Fig. 6, for XY54 and LR group lines, apparent staining signals were observed in both the root meristematic zone and elongation zone, however, apparent signals of NBT staining were observed only in the root meristematic zone of J411 and lines in the SR group (Fig. 6A). These results suggested that O_2_.^-^ was distributed abundantly both in the meristematic and elongation zones in XY54 and LR group lines but only in the meristematic zone of J411 and SR group lines. The results of DAB staining for H_2_O_2_ were similar to those of NBT staining (Fig. 6B).

### Real-time PCR analysis of peroxidase genes in different zones of root tip

**Figure 7:**
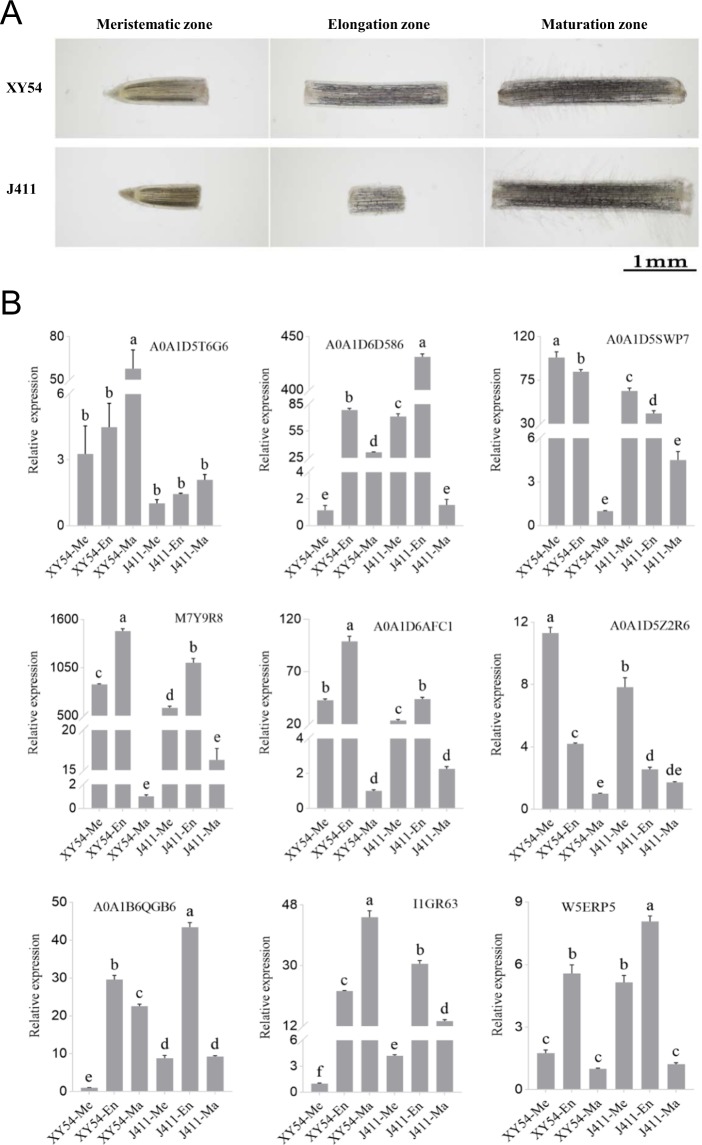
Relative mRNA expression analysis of differentially expressed peroxidase genes in the meristematic, elongation and maturation zones of root tips using RT-qPCR. ‘Me’ represent meristematic zone; ‘En’ represent the elongation; ‘Ma’ represent maturation zone. The lowest expression level in the samples was set as 1.0. Columns marked with different lowercase letters indicate that the difference between them (in gene expression levels) is significant according to Duncan’s multiple range test (n = 3, p < 0.05).

We totally identified nine peroxidases in the DEPs. Compared with the SR group, six of them were up-regulated and three were down-regulated in the LR group (Supplementary Table S4). As O_2_.^-^ and H_2_O_2_ were differentially distributed in the meristematic and elongation zones between the long root genotype and short root genotype (Fig. 6), and peroxidases can regulate ROS production^33^, we further analysed the expression levels of these peroxidase genes in the meristematic, elongation and maturation zones of XY54 and J411 root tips (Fig. 7A). Results showed that the expression levels of different peroxidase genes varied greatly in different root apical zones and different varieties (Fig. 7B). Among the nine peroxidase genes, five of them had significantly higher expression levels in the elongation zone than in the meristematic and maturation zones, while two of them had significantly higher expression levels in the meristematic zone (A0A1D5Z2R6, A0A1D5SWP7) than in the elongation and maturation zones (Fig. 7B). In addition, the other two peroxidase (A0A1D5T6G6 and I1GR63) genes had the highest expression levels in the maturation zone in the long root plants XY54, but not in the short root plants J411. In XY54, the expression levels of four peroxidase (A0A1D5SWP7, M7Y9R8, A0A1D6AFC1 and A0A1D5Z2R6) genes in both the meristematic and elongation zones were significantly higher than those in J411 (Fig. 7B). While the expression levels of another four peroxidase genes (A0A1D6D586, A0A1B6QGB6, I1GR63 and W5ERP5) in both the meristematic zone and elongation zone of J411 were significantly higher than those of XY54 (Fig. 7B).

## Discussion

Deeper roots contribute to plant production and survival under water and nitrogen limiting conditions^36–38^. However, the molecular mechanisms controlling root growth in wheat remain unclear. Here, an integrated comparative proteome study was performed to explore this question. Eighteen DEPs including the nine differentially expressed peroxidases were selected to evaluate the correlation of protein expression with their corresponding mRNA level using real-time PCR. Eleven genes had closely matched mRNA levels with their translation products while the expression levels of the other seven genes exhibited poor correlations with their changes in protein abundance (Fig. 4B). One possible reason is that post-transcriptional, translational and post-translational regulation may occur in the expression of those poorly correlated genes^39–41^. Another possible reason is that the peroxidase genes belong to a huge gene family in hexaploid wheat, and some peroxidase members have a high degree of homology. Therefore, the poor correlation may be due to the lack of specificity for the genes encoding the identified proteins.

In this work, 80 DEPs associated with the regulation of primary root growth were identified, including two plant steroid biosynthesis related proteins (CYP51 and DWF1) (Fig. 3; Supplementary Table S4). Plant steroid biosynthesis pathway was significantly enriched in the up-regulated DEPs (Fig. 2C). Coincidentally, TaTRIP1, a negative regulator in BR signalling, has been shown to be up-regulated in short-root wheat varieties^28^. Both CYP51 and DWF1 are essential for the biosynthesis of plant steroids. DWF1 catalyses the formation of CR, while CR is the first molecule specifically entering BR biosynthetic pathways^42^. Moreover, the expression levels of *TaCYP51* and *TaDWF1*, and four other genes (*TaDWF4*, *TaDWF6*, *TaDWF7* and *TaCYP90D1*) involved in BR biosynthesis pathway also exhibited significantly up-regulated pattern in XY54 and the LR group compared with J411 and the SR group (Fig. 5). These results indicated that the primary root growth was possibly promoted by elevated BR biosynthesis. Brassinolide (BL) is reported as a potent BR in plants^43,44^, so we measured the content of endogenous BL in LRM and SRM using high performance liquid chromatography (HPLC)-MS/MS. A small amount of BL was detected in the LRM, but not in the SRM (below the detection limit; data not shown). We repeated the experiment and obtained a similar result. As a matter of fact, similar results have also been reported in another gramineous plant, rice^45,46^. Therefore, it is likely that some other sterols (e.g. castasterone) other than BL act as the main active BRs in wheat. However, further studies are needed to verify this point.

Noticeably, among the 80 DEPs identified in this study, nine of them were annotated as class III peroxidases of which six were up-regulated and three were down-regulated (Fig. 3; Supplementary Table S4). Class III peroxidases belong to a large multigene family in plants. 73 and 138 genes encoding class III peroxidase genes have been identified in *Arabidopsis* and rice, respectively^47–49^. Class III peroxidases can catalyse the reduction of H_2_O_2_ by taking electrons to various donor molecules (such as lignin precursors or secondary metabolites) in the regular peroxidative cycle^49,50^. Real-time PCR analysis of the peroxidase genes in different zones of root tips showed that some peroxidase genes had higher expression in the meristematic zone, while some others in the elongation zone. The results suggest that different members of the peroxidase gene family may play roles in different zones of root tips. Furthermore, the expression levels of different peroxidase genes also varied greatly in the root tips of XY54 and J411 (Fig. 7B). It is not clear why some differentially expressed peroxidases were highly expressed in the long root genotype, while others were expressed in the short root genotype. One possible reason is that different members of this large gene family may have functional differentiation. Actually, previous studies have shown that peroxidases can not only catalyze the cleavage of H_2_O_2_, but also has oxidase activity, which can also catalyze the formation of O_2_.^-^ and H_2_O_2_ in the presence of NAD(P)H^51–53^. Thus, the regulation of peroxidases on reactive oxygen species (ROS) is very complicated. As ROS balance has an important role in maintaining root meristem activity^54^, different peroxidases may play different roles in mediating the balance of ROS between the root meristematic zone and elongation zone. In our study, ROS (O_2_.^-^ and H_2_O_2_) accumulated both in the root meristematic zone and elongation zone of XY54 and LR, while accumulation was observed only in the root meristematic zone of J411 and the SR group (Fig. 6). The differential accumulation of ROS between the LR and SR groups may lead to different meristem activity and thus different PRL and MRL. BRs are important regulators of many important biological processes^55^. Ca^2+^ and ROS can act as second messengers to function in BR-regulated biological processes^56–59^. In addition, MAPK cascades also control ROS production in BR-mediated responses^60,61^. Such evidence suggests that ROS may be an integration node for BR signalling pathway with root developmental pathways.

BRs have been reported to regulate RAM size by promoting cell cycle progression in *Arabidopsis*^62,63^. Overexpression of a negative regulatory gene of wheat BR signalling, *TaTRIP1*, in *Arabidopsis*, reduces *Arabidopsis* root meristem size, and hence PRL^28^. Plants treated with BR inhibitor have reduced ROS levels, whereas exogenous BRs induce moderate ROS accumulation^64^. Moreover, BR biosynthesis pathway was elevated in plants of the LR group compared with the SR group (Figs 2C and 5). Taken together, the regulation of wheat primary root growth is closely related to BR biosynthesis pathway and BR-mediated ROS distribution. Based on our experimental evidences and previous reports, we proposed a possible regulatory mechanism: the BR-mediated ROS accumulation and distribution in root tips determined root meristem size, which determines the growth rate of primary roots. And the differential accumulation and distribution of ROS in the root tips of different genotypes may be due to the differential expression of a number of peroxidases.

## Materials and Methods

### Plant materials and evaluation of root morphology

The LR group and SR group were selected based on previous studies from a RIL population^25,29^. The plants were grown in a greenhouse with six replications each. Methods for seed sterilization, germination and the growth conditions of wheat plants were as previously described by Ren *et al*.^25^. 12 d after transfer, the root phenotype of each line was investigated. Roots of each line from the LR and SR groups were mixed, respectively (LRM and SRM). The reason for choosing this stage is that the primary root keeps elongate in the long root genotype, but the elongation of the short root genotype almost stops after two weeks. The root samples of LRM, SRM and their parents (XY54 and J411) were quick-frozen in liquid nitrogen and stored in a −80 °C freezer for subsequent protein and RNA extraction. Three biological replicates were analysed to minimize experimental error.

### Protein extraction, digestion and iTRAQ labeling

Protein extraction was conducted according to the method described by Thiellement *et al*.^65^. Protein digestion was conducted using the FASP procedure^66^. Protein samples were digested overnight in 50 μL trypsin solution at 37 °C, and then labelled with iTRAQ reagents based on the manufacturer’s instructions (Applied Biosystems, USA). Three biological replicates were performed. Four eight-plex iTRAQ sets was used to label the samples. XY54-1, XY54-2 and XY54-3 were labeled with 113 of set 1, set 2 and set 3, respectively; J411-1, J411-2 and J411-3 were labeled with 114 of set 1, set 2 and set 3, respectively; LRM-1, LRM-2 and LRM-3 were labeled with 113, 114 and 115 of set 4, respectively; SRM-1, SRM-2 and SRM-3 were labeled with 116, 117 and 118 of set 4, respectively. The remaining labels of each set together with the 113 and 114 of the first three sets were used for another study.

### SCX fractionation and LC-ESI-MS/MS analysis

AKTA Purifier system (GE Healthcare, USA) was used for the SCX (strong cationic exchange) chromatography to fractionate the iTRAQ labelled peptides as described previously with minor modifications^67–69^. The elution was surveyed using the absorbance at 214 nm. In total, 30 fractions were collected (1 fraction/min) and merged into 10 parts, and each part were then desalted and reconstituted in 40 µl 0.1% (v/v) trifluoroacetic acid. A Q Exactive mass spectrometer coupled to an Easy-nLC (Proxeon Biosystems) was used for the LC ESI-MS/MS (liquid chromatography-electrospray ionization tandem MS) analysis according to previous reports^67–69^.

### Protein identification and quantification

The LC-MS/MS analysis were performed in positive ion mode. The most abundant precursor ions were chosen from the survey scan (300–1800 m/z) for HCD fragmentation using the data-dependent TOP10 method; the peptide recognition mode was run in the instrument^69^. Raw data were collected by Proteome Discoverer 1.4 software (Thermo Fisher Scientific), and fragmentation spectra were identified using the MASCOT 2.2 search engine running against the uniprot_Poaceae database (http://www.uniprot.org). The parameters for protein identification were set as follows: missed cleavages ≤2, the mass tolerance was 20 ppm for precursor ions and 0.1 Da for fragmented ions, FDR ≤1%. For the quantitative analysis of the identified proteins, all peptide ratios were normalized by the median protein ratio using Proteome Discoverer 1.4. After the normalization, the median protein ratio is 1.0. The relative expression values of each protein were normalized using the median ratio in Mascot^69–71^. Only those proteins detected in at least two replicates were used for the following DEP analysis. Protein species with a ratio fold change greater than or equal to 1.2 (p < 0.05) were defined as DEPs^40,72^. Here, we selected a relatively lower ratio (1.2-fold change) because the DEPs involved in the regulation of wheat primary root growth were defined by two steps to reduce the possible interference of the genetic background of XY54 and J411. Only those DEPs that coexist in XY54-J411 and LRM-SRM comparisons were considered to be the final DEPs.

### Bioinformatics analysis

NCBI BLAST + client (ncbi-blast-2.2.28 + -win32.exe) and BLAST2GO were used for GO annotation^73^. GO enrichment analysis was applied based on Fisher’s exact test using all the quantified protein annotations as a background dataset (p < 0.05). The protein pathway analysis was performed using KOBAS 3.0 (http://kobas.cbi.Pku.edu.cn./anno_iden.php). Functional categories and pathways with a corrected p-value < 0.05 were considered as significant. Cluster3.0 (http://bonsai. hgc.jp/~mdehoon/software/cluster/software.htm) together with the Java Treeview software (http://jtreeview.sourceforge.net) were used to perform hierarchical clustering analysis.

### Quantitative Real-time PCR

The meristematic, elongation and maturation zones of root tips were dissected by a shaving blade under a microscope (OLYMPUS BX-53, Japan). The whole root samples and different root apical zone samples were quick-frozen in liquid nitrogen and stored in a −80 °C freezer for RNA extraction. Total RNA of each sample was isolated using TRIzol Reagent (Invitrogen, USA) with genomic DNA removed using DNase I (Promega, USA). Each total RNA sample was used to synthesize the first strand of cDNA using the GoScript Reverse Transcription System (Promega, USA) following the manufacturer’s instructions. Primer 5.0 software (Premier, Canada) was used to design real-time PCR primers (Supplementary Table S6). SYBR Green PCR Real Master Mix (Tiangen, Beijing, China) was used for real-time PCR analysis on a Thermal Cycler CFX96 Real-Time System (Bio-Rad, USA) according to the instructions. The relative expression levels of target genes were computed using the 2^−ΔΔCT^ method, with *TaActin* as the reference gene.

### DAB and NBT staining

*In situ* H_2_O_2_ and O_2_.^-^ was stained using DAB (3,3′-diaminobenzidine) and NBT (nitroblue tetrazolium) respectively according to previous descriptions^35,74^. The results of NBT and DAB staining were photographed using a camera (Canon EOS 70D, Japan) mounted on a microscope (OLYMPUS BX-53, Japan).

### Statistical analysis

The mean values and standard errors were analysed using Excel 2017 and IBM SPSS statistics 21 software. The statistical significance of differences was analysed using Duncan’s multiple range test for all traits evaluated and gene expression levels, with a p-value < 0.05 considered significant.

## Supplementary information

Supplementary Information

Dataset 1

## Data Availability

MS data in this study has been deposited in the iProX repository (ww.iprox.org) with identifier PX0001149000/PXD008793.
