# Supplementary material for: Comparative Proteomic Analysis Provides Insights into the Regulatory Mechanisms of Wheat Primary Root Growth
Source: Sci Rep. 2019 Aug 13;9:11741. doi: 10.1038/s41598-019-47926-7 (PMC6692329; doi:10.1038/s41598-019-47926-7)
Supplement: Supplementary file 1 — Supplementary Information [file 41598_2019_47926_MOESM1_ESM.pdf]

**Comparative Proteomic Analysis Provides Insights into the Regulatory Mechanisms of Wheat Primary Root Growth**

Le Li[1,2,3†], Yanhua Xu[1,2,3,4†], Yongzhe Ren[1,2,3*], Zhanyong Guo[1,2,3], Jingjing Li[1,2,3], Yiping Tong[5], Tongbao Lin[1,2,3*] and Dangqun Cui[1,2,3*]

[1]College of Agronomy, Henan Agricultural University, Zhengzhou, China.

[2]State Key Laboratory of Wheat and Maize Crop Science, Henan Agricultural University, Zhengzhou, China.

[3]Collaborative Innovation Center of Henan Grain Crops, Henan Agricultural University, Zhengzhou, China.

[4] Shangqiu Normal University, Shangqiu, China.

[5] State Key Laboratory for Plant Cell and Chromosome Engineering, Institute of Genetics and Developmental Sciences, Chinese Academy of Sciences, Beijing, China. [†]Yanhua Xu and Le Li contributed equally to this work.

**Supplementary Information**

Supplementary Table S1 List of reliable detected proteins for quantitative analysis.

Supplementary Table S2 List of differentially expressed proteins (DEPs) in the XY54-J411 comparison.

Supplementary Table S3 List of differentially expressed proteins (DEPs) in the long root mixture-short root mixture (LRM-SRM) comparison.

Supplementary Table S4 Differentially expressed proteins (DEPs) involved in the regulation of primary root growth.

Supplementary Table S5 List of significantly enriched pathways in the up-regulated differentially expressed proteins (DEPs).

Supplementary Table S6 Primers used for real-time PCR.
